# TrpA1 activation in peripheral sensory neurons underlies the ionic basis of pain hypersensitivity in response to vinca alkaloids

**DOI:** 10.1371/journal.pone.0186888

**Published:** 2017-10-30

**Authors:** Nina Boiko, Geraldo Medrano, Elizabeth Montano, Nan Jiang, Claire R. Williams, Ngonidzashe B. Madungwe, Jean C. Bopassa, Charles C. Kim, Jay Z. Parrish, Kenneth M. Hargreaves, James D. Stockand, Benjamin A. Eaton

**Affiliations:** 1 Department of Cellular and Integrative Physiology, University of Texas Health Sciences Center at San Antonio, San Antonio, Texas, United States of America; 2 Department of Biology, University of Washington, Seattle, Washington, United States of America; 3 Verily, South San Francisco, California, United States of America; 4 Department of Endodontics, University of Texas Health Sciences Center at San Antonio, San Antonio, Texas, United States of America; Indiana University School of Medicine, UNITED STATES

## Abstract

Chemotherapy induced peripheral neuropathy (CIPN), a side effect of many anti-cancer drugs including the vinca alkaloids, is characterized by a severe pain syndrome that compromises treatment in many patients. Currently there are no effective treatments for this pain syndrome except for the reduction of anti-cancer drug dose. Existing data supports the model that the pain associated with CIPN is the result of anti-cancer drugs augmenting the function of the peripheral sensory nociceptors but the cellular mechanisms underlying the effects of anti-cancer drugs on sensory neuron function are not well described. Studies from animal models have suggested a number of disease etiologies including mitotoxicity, axonal degeneration, immune signaling, and reduced sensory innervations but these outcomes are the result of prolonged treatment paradigms and do not necessarily represent the early formative events associated with CIPN. Here we show that acute exposure to vinca alkaloids results in an immediate pain syndrome in both flies and mice. Furthermore, we demonstrate that exposure of isolated sensory neurons to vinca alkaloids results in the generation of an inward sodium current capable of depolarizing these neurons to threshold resulting in neuronal firing. These neuronal effects of vinca alkaloids require the transient receptor potential ankyrin-1 (TrpA1) channel, and the hypersensitization to painful stimuli in response to the acute exposure to vinca alkaloids is reduced in *TrpA1* mutant flies and mice. These findings demonstrate the direct excitation of sensory neurons by CIPN-causing chemotherapy drugs, and identify TrpA1 as an important target during the pathogenesis of CIPN.

## Introduction

Chemotherapy induced peripheral neuropathy (CIPN) is a common dose-limiting side effect of many anti-cancer drugs, including vinblastine and vincristine, that is associated with a severe peripheral pain syndrome [1]. There is strong evidence suggesting that the pain associated with CIPN is the result of the anti-cancer drugs acting directly upon the peripheral sensory nociceptors [2–8]. In addition, the pain resulting from these drugs occurs soon after chemotherapy treatment begins suggesting that altered sensory function is an early event during the pathogenesis of CIPN. Although a number of cellular pathologies have been observed in somatosensory neurons after prolonged exposure to anti-cancer drugs, the early formative events associated with the pathogenesis of CIPN remain unclear [2,7,9–12].

One barrier that has impeded the determination of the neuronal mechanisms underlying chemotherapy pain is the functional heterogeneity of sensory neurons within the mammalian dorsal root ganglion (DRG) [7,13–17]. For example, recent studies have found that the chemotherapy drug paclitaxel changes the intrinsic excitability of a subset of sensory neurons making determination of the molecular and cellular mechanisms underlying this effect of paclitaxel challenging [7]. The class IV dendrite arborization (C4da) sensory neurons in *Drosophila melanogaster* larvae are polymodal sensory neurons required for response to a broad range noxious stimuli during the larval stage of development [18]. These neurons were initially characterized based upon their stereotyped dendritic morphology but have been shown to be critical sensory neurons endowing the larvae with a surprisingly large repertoire of sensory modalities [18–20]. The larval C4da neurons faithfully mimic the properties of mammalian DRG nociceptors including the responsiveness to multiple sensory modalities including temperature, chemical, and mechanical stimuli [21–23]. Importantly, C4da-specific driver lines allow for experimental isolation of this specific class of sensory neurons for subsequent cellular analyses including high resolution imaging and electrophysiological interrogation [19,24–26]. The similarities with mammalian sensory neurons and the experimental tractability of the *Drosophila* model organism make the C4da neuron a powerful system for the study of pain during CIPN.

In addition to function, the sensory signal transduction machinery is highly conserved between *Drosophila* and mammals including the Transient receptor potential (Trp) family of ion channels [27]. Members of this large class of ion channels have been implicated in a wide range of sensory perception including noxious chemical, mechanical, and thermal sensation [28,29]. The TrpA1 family of channels that play a critical and conserved role in peripheral sensory neurons mediating avoidance responses to a broad range of noxious stimuli including chemicals [30–40]. Despite the large number of studies implicating TrpA1 channels in chemical nociception, to date only a few studies have investigated directly a role for TrpA1 channels during CIPN and none of these studies have demonstrated a direct effect of vinca alkaloids on the activation of this channel [8,41–44]. Here we show in both flies and mice that the acute exposure of peripheral sensory neurons to vinca alkaloid anti-cancer drugs results in a robust pain syndrome that is characterized by a rapid increase in the sensitivity to both mechanical and thermal stimuli. Mutational analyses in both mice and flies demonstrates that this effect of vinca alkaloids requires TrpA1 revealing an ancient and conserved molecular mechanism of these anti-cancer drugs on sensory function. Patch-clamp analysis of *Drosophila* C4da sensory neurons reveals that vinca alkaloids rapidly activate a depolarizing inward current that is sensitive to TrpA1 antagonists and absent in *dTrpA1* mutant neurons. These data demonstrate that vinca alkaloids can directly activate sensory neurons in a TrpA1-dependent fashion and support the model that activation of TrpA1 in sensory neurons represents an early event during the pathogenesis of CIPN.

## Materials and methods

### Drosophila stocks and husbandry

Drosophila stocks were maintained on standard food (Bloomington recipe) supplemented with dry yeast at 25C and a 12-hour light/dark cycle. The *ppk1-Gal4*, *UAS-mCD8-GFP* fly line was a gift from Darren Williams (King’s College London, UK). *dTrpA1*^*1*^, *painless*, and *UAS-dTrpA1* (Stock# 26263) transgene stocks were obtained from the Bloomington stock center. The *painless* mutant was analyzed as a trans-allelic combination of *pain*^*EP2451*^ and *pain*^*EP2251*^. The *ppk-Gal4*, *UAS-Red Stinger*, *yw;; ppk-CD4tdTomato*, and the *hs*::*Flp; ppk-Gal4*, *UAS-Frt-stop-Frt-mCD8-GFP* stocks are part of the Parrish lab stocks [45]. The genotype for the TrpA1 rescue line was the following: *w*^*1118*^; *ppk1-Gal4*, *UAS-mCD8-GFP/UAS-dTrpA1; dTrpA1*^*1*^*/dTrpA1*^*1*^.

### Drosophila primary cell culture

The primary neuronal cell culture was prepared from midgastrula stage embryos as described previously [25,26]. In brief, 3–4 hour-old embryos were collected and dechorionated with 50% commercial bleach. The contents of 3–4 embryos were dispersed onto a glass coverslip and maintained in 5% CO_2_ at 23°C for 2–3 days with serum-free Ham's F12/DME media plus 20 mM HEPES and 2.5 mM L-glutamine. The culture media was supplemented with 50 μg/mL insulin, 20 ng/mL progesterone, 100 μg/mL transferrin, 30 nM selenium, and 100 μM putrescine. Neurons in these midgastrula stage embryo cultures arise from neuroblast precursors. For electrophysiology experiments cultured neurons were used 2–3 days after seeding and for up to one week.

### Electrophysiology

C4da sensory neurons were identified using epi-fluorescence for GFP expression, as driven by *ppk1-Gal4*,*UAS-mCD8-GFP*. Action potentials from these neurons were recorded under whole-cell current-clamp conditions as described previously [25,26]. For these experiments, the pipette solution was (in mM) 120 K-gluconate, 20 NaCl, 0.1 CaCl_2_, 1 EGTA, and 10 HEPES (pH was adjusted to 7.2 with KOH). Macroscopic current recordings from C4da neurons were performed under voltage-clamp conditions at a holding potential of -60 mV as described previously [25,26]. The pipette solution for these experiments was (in mM) 120 Cs-gluconate, 20 NaCl, 0.1 CaCl_2_, 1 EGTA, and 10 HEPES (pH was adjusted to 7.2 with CsOH). The bath solution for both current- and voltage-clamp recordings was (in mM) 140 NaCl, 5 KCl, 1 CaCl_2_, 10 D-glucose, and 10 HEPES, (pH was adjusted to 7.4 with NaOH). Action potentials and currents were filtered at 1 kHz and acquired at 2 kHz with an Axopatch 200B (Molecular Devices, Sunnyvale, CA) interfaced via a Digidata 1322A (Molecular Devices) to a PC running the pClamp 10.5 software suite (Molecular Devices). Resistances of recording pipettes ranged from 10 to 13 MΩ. For all recordings, n = 5–12 cells for each condition collected from 2–5 separate platings.

### Fly nociception assays

The mechanical nociception was tested using a standard protocol [21,26,46,47]. Briefly, third-instar larvae were transferred to standard food supplemented with chemotherapy drugs at indicated concentration for 1 hour to overnight. For antagonist studies, vinblastine-containing food was supplemented with HC-030031 at 100 uM. For all conditions, only actively moving larvae were exposed to a single transient mechanical stimulus applied to the abdominal region with calibrated Von Frey filaments. Positive responses were scored only when larvae performed at least one 360° rotation around the anterior-posterior axis. Values represent assays performed with von frey fibers rated between 45–55 mN which result in ~50% response rates in control larvae. Larvae are assayed in groups of 20 with control groups interspaced with treatment groups during the analyses. Average response values are determined by averaging the average group values obtained from at least 8–10 groups of treated or control larvae. Values presented in figures are final average treatment values normalized to final average vehicle values. For thermal hyperalgesia, 30 treated larvae were arrayed on glass plates and videotaped during a 37C challenge for 2 minutes. Videos were retrospectively inspected between 30 seconds and 1 minute for the nocifensive rolling behavior assayed above. Control is collected and data analyzed as described for mechanical nociception.

### Live imaging of C4da neurons

Embryos (*yw;; ppk-CD4tdTomato*) were collected for 24 h on yeasted cornmeal molasses plates, aged for 72 h, and transferred to fresh plates (with or without 10 μM vinblastine) for 1 h of feeding prior to imaging. At the appropriate time, a single larva was mounted in 90% glycerol under coverslips sealed with vacuum grease and imaged on a Leica SP5 microscope with a 40X 1.25 NA lens. For quantitation of dendrite phenotypes, image stacks of dendrites in segments A3-A4 were captured from eight to ten larvae. Two-dimensional (2D) projections of Z-stacks were used for computer-assisted dendrite tracing with Neurolucida (MBF Bioscience), and total dendrite lengths were measured using the traces.

### Imaging of C4da axon terminals

Single class IV md (C4da) neuron clones were generated by using a FLP-out approach [48]. Briefly, 1^st^ instar larvae of the genotype *hs*::*Flp; ppk-Gal4*, *UAS-Frt-stop-Frt-mCD8-GFP* were heat shocked at 37 C for 30 min to induce FLP recombinase expression, resulting in stochastic labeling of C4da neurons. Larvae with single C4da clones were selected at 72 h after egg laying and fed with 10 μM vinblastine at 25 C for 1 h. Larvae ventral nerve cords (VNCs) were dissected and immunolabeled with mouse-anti-GFP (1:100, Invitrogen) and Cy2-conjugated secondary antibodies (1:200, Jackson Immunoresearch). Immunostained VNCs were imaged on a Leica SP5 microscope with a 40X 1.25 NA lens. Lengths of axon terminals were measured using ImageJ.

### Mouse nociception assay

The Institutional Animal Care and Use Committee of the University of Texas Health Science Center at San Antonio approved all protocols. Male C57 mice or DTRPA1-/- mice (RRID:MGI:3625358; Jackson Labs; Sacramento, CA, USA) were used for all studies. Animals were housed for at least 7 days prior to the experiments to allow for acclimation. All observers were blinded to treatment allocation, with n = 7 per group. Paw-withdrawal latencies to radiant heat were tested 30 minutes after injection as previously described [49]. A dynamic plantar anesthesiometer was used to measure mechanical allodynia and paw-withdrawal were measured 15 minutes after injections [50]. Spontaneous nocifensive behavior was measured as the duration (s) spent flinching or licking the injected hind paw during a 15 min period after intraplantar injection as previously defined [51].

### Calcium imaging

Calcium imaging on primary cultures of Trigeminal ganglion (TG) neurons was performed as previously described [52] Briefly, TG was gently triturated and cells from six ganglia were plated on poly-d-lysine/laminin-coated coverslips for 24 hours. Neurons were loaded with 1μM of the cell permeable calcium sensitive dye, FURA 2AM (Molecular Probes, Eugene, OR), in presence of 0.01% Pluronic (Molecular Probes) for 30min at 37°C. Coverslips containing cells were placed in a chamber with constant infusion of external buffer (SES) of the following composition (in mM): 140 NaCl, 5 KCl, 2 CaCl_2_, 1 MgCl_2_, 10 glucose and 10 Hepes, pH 7.4. at 37°C. Fluorescence images of 340 and 380 excitation wavelengths were collected for 200ms, each 5s throughout the experiment, analyzed and the 340/380 ratio calculated by the MethaFluor Software (MethaMorph, Web Universal Imaging Corporation, Downingtown, PA). Cells were preincubated with vehicle or 10uM HC-030031 for 15 minutes. Cells were then imaged for baseline and then challenged with vehicle, vinblastine (10uM and 100uM), or vinblastine + HC-030031 for 3min, after which responsive cells were allowed to recover for approximately 1–2min. Trp-V1 expressing neuron types were identified by applying capsaicin (30nM at 40s) to the cells at the end of the experiment. The net change in internal Ca^2+^ was calculated by subtracting the basal Ca^2+^ levels (mean value collected for 30s prior to agonist addition) from the peak Ca^2+^ levels achieved after exposure to the agonists. Under these condition, calcium accumulation ratio changes below 0.03 are considered noise (indicated with a gray line).

### Preparation of isolated mitochondria

Mitochondria were isolated from adult CFW (Swiss Webster) male standard mice (Charles River Labs, Wilmington, MA), 8–16 weeks old. Mice were anesthetized by intraperitoneal injection of pentobarbital (60 mg/kg), and heparin (200 UI/kg) was used to prevent blood coagulation. Hearts were surgically removed and immediately arrested in cold (4°C) Krebs Henseleit bicarbonate buffer (KH) solution (mM): glucose 11, NaCl 118, KCl 4.7, MgSO_4_ 1.2, KH_2_PO_4_ 1.2, NaHCO_3_ 25 and CaCl_2_ 3, pH 7.4. Myocardial sections (approximately 0.15–0.22 g) were placed in isolation buffer A (mM): sucrose 70, mannitol 210, EDTA 1 and Tris-HCl 50, pH 7.4. The tissue was finely minced and homogenized in the same Buffer A (0.1 g of tissue/ml of buffer). The homogenate was centrifuged at 3,000 rpm for 3 minutes in a Galaxy 20R centrifuge (VWR, Radnor, PA); the supernatant was centrifuged at 13,000 rpm for 10 minutes. The mitochondrial pellet was resuspended in isolation Buffer B (mM): sucrose 150, KCl 50, KH_2_PO_4_ 2, succinic acid 5 and Tris/HCl 20, pH 7.4). Protein concentration was estimated using the Bradford method assay kit (Bio-Rad, Hercules, CA).

### Ca^2+^-induced mitochondrial permeability transition

The installation of mitochondrial permeability transition pores was assessed following *in vitro* Ca^2+^ overload. Free Ca^2+^ concentration outside the mitochondria was recorded using 0.1 μM calcium green-5N (Thermo Fisher) which binds reversibly to Ca^2+^, using excitation and emission wavelengths set at 500 and 530 nm, respectively. Isolated mitochondria (500 μg of protein) were suspended in 2 ml isolation Buffer B with 10μM vinblastine or vehicle and pre-incubated for 90 seconds in a spectrofluorimeter (Hitachi F-2710) set at 30°C. CaCl_2_ pulses (10 μmoles or 10 μL of 1 mM stock solution) were applied every 60 sec to the cuvette leading to 20 nmol Ca^2+^ (per mg of protein) without the mitochondrial uptake. The Ca^2+^ pulses induce a peak of extra-mitochondrial Ca^2+^ concentration that returns to near-baseline levels as Ca^2+^ enters the mitochondrial matrix via uptake by the Ca^2+^ uniporter. With increasing calcium loading, the extra-mitochondrial Ca^2+^ concentration starts accumulating, thereby reflecting a lower capacity for mitochondrial Ca^2+^ uptake. This is followed by a sustained Ca^2+^ increase, indicating a massive release of the mitochondria Ca^2+^ by the mPTP opening. The Ca^2+^ retention capacity (CRC) was defined as the amount of Ca^2+^ required to trigger this massive Ca^2+^ release which is used here as an indicator of the mPTP sensitivity to Ca^2+^. CRC is expressed as nmol of CaCl_2_ per mg of mitochondrial protein. Analysis was performed on mitochondria isolated from 7 independent hearts.

### RNA-seq of C4da neurons

Five whole larvae and four samples of one hundred C4da neurons each were isolated and RNA-Seq libraries were prepared as described previously [53,54]. Briefly, third instar larvae (*ppk-Gal4/+; UAS-Red Stinger/+)* were dissociated and C4da neurons were isolated by flow cytometry into RNAqueous lysis buffer (ThermoFisher). For whole larva samples, individual larvae of the same genotype were ground with a pipette tip and then snap frozen in lysis buffer on dry ice. RNA was isolated using the RNAqueous Micro Kit and concentrated to 1 μl (C4da neuron samples) or diluted to 0.2 ng/ μl (whole larvae samples). Pre-amplified cDNA libraries were generated with template-switching reverse transcription [55,56], using the SMARTer Ultra-low input kit. cDNA was fragmented, barcoded and amplified using the Nextera XT DNA kit, and libraries were pooled and purified using AMPure XP beads. Samples were sequenced as 51 base single end reads on a HiSeq 2500 running in high-output mode at the UCSF Center for Advanced Technology, with read depths ranging from 1.2 to 11.4 million reads. Reads were demultiplexed with CASAVA (Illumina) and read quality was assessed with FastQC (http://www.bioinformatics.babraham.ac.uk/projects/fastqc/). Reads were aligned to the *D*. *melanogaster* transcriptome, FlyBase genome release 6.10, using STAR version 2.5.2b [57] with the option ‘—quantMode TranscriptomeSAM’. Transcript expression was modeled from these STAR alignments using Salmon in alignment-based mode. Gene expression was determined by summing the transcript expression, in tpm, for all transcripts of a given gene. The raw sequencing reads and gene expression estimates are available in the NCBI Sequence Read Archive (SRA) and in the Gene Expression Omnibus (GEO) under accession number GSE99711.

### Data and statistics

Data are reported as the average ± SEM. Significance determined using Student’s T-test for pairwise comparisons and one-way ANOVA with a Bonferroni correction for all multiple comparisons. All statistical analyses were performed using Prism 6 (Graphpad).

## Results

### Feeding Drosophila larvae vinca alkaloids rapidly generates hypersensitivity to mechanical and thermal stimuli

**Fig 1:**
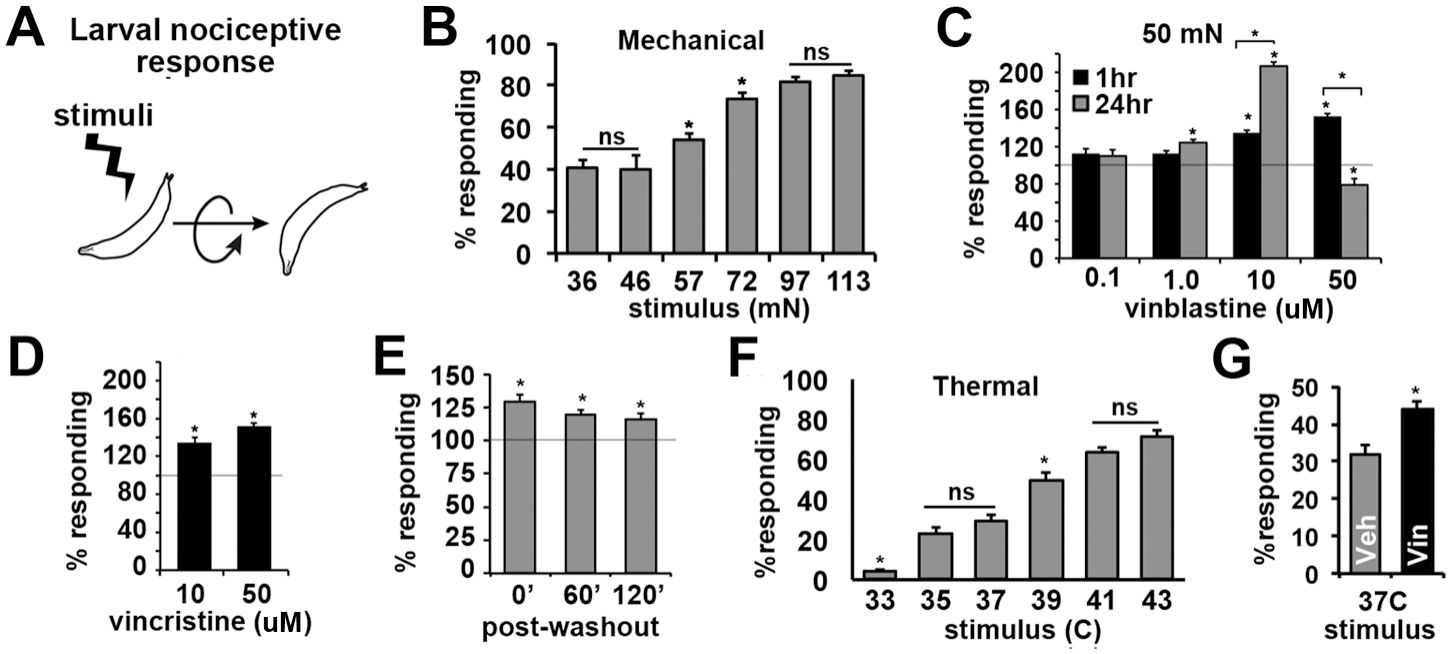
Vinca alkaloids generate neuropathic pain in *Drosophila* larvae. **A**, Nocifensive stimuli to larvae elicit a stereotypical rolling response. **B**, Force-response curve for indicated forces. Graphs represent the average values for responding larvae. **C-E**, Increased responses of larvae to mechanical stimulation (50-60mN) after feeding vinblastine (**C**) or vincristine (**D**) at indicated concentrations for 1 hr (black bars) or 24 hrs (gray bars) versus vehicle controls. (**E**) Increased responsiveness persists after larvae are removed from drug. In all drug treatment studies, average treatment values were normalized to vehicle controls assayed in parallel. Gray line indicates vehicle (100%) values. **F**, Temperature-response curve for indicated temperatures. Graphs represent the average values for responding larvae. **G**, Increased response of larvae to thermal stimulation (37°C) after 1 hour feeding of vinblastine (50 μM). Graphs represent the average values for responding larvae. Error bars = SEM, n = 160–200 larvae assayed in groups of 20 per each condition. Significance determined by ANOVA except for drug treatments where significance was determined using a Student’s T-test for drug vs. vehicle for each condition except where indicated (*p<0.05). † indicate significant differences determined by ANOVA analysis (p<0.01).

*Drosophila* larvae exhibit a characteristic nocifensive behavioral response to mechanical and thermal stimuli (Fig 1A) [21,26,46]. This nocifensive rolling behavior is dependent upon the normal function of the C4da neuron [58]. As shown in Fig 1B, there is a dose-response relation between mechanical force and the number of responding larvae. We observe that *Drosophila* larvae fed vinblastine have increased responses to sub-threshold mechanical stimuli (Fig 1C). This effect of vinblastine is dependent upon both concentration and treatment time (Fig 1C). Feeding larvae high concentrations (50 uM) for 24 hours reduces sensory function and increases lethality (Fig 1C). Vincristine, an analogue of vinblastine, similarly increases sensitivity to mechanical stimuli in a dose-dependent manner (Fig 1D). This effect of vinca alkaloids on pain sensitivity is rapid appearing within the first hour following exposure to 10 uM vinblastine (Fig 1C and 1D, black bars) and persisting for up to two hours following removal of larvae from food containing vinblastine (Fig 1E). Larvae respond to noxious temperatures with a similar C4da neuron-dependent rolling behavior that shows a strict temperature-dependence (Fig 1F). Similar to its effect on mechanical nociception, we find that treatment with vinblastine causes thermal hyperalgesia (Fig 1G). These observations demonstrate that acute exposure to vinca alkaloids generates increased pain sensitivity in *Drosophila* that is not modality specific and persists in the presence and absence of drug.

**Fig 2:**
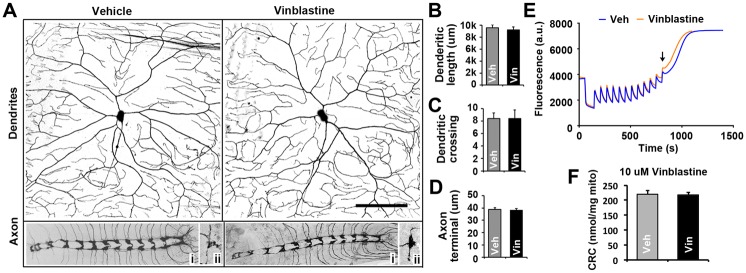
Acute exposure to vinca alkaloids has no effect on C4da sensory neuron morphology or mitochondrial toxicity. **A**, Confocal images of dendritic arbors, axonal projections (i), and axonal nerve terminals (ii) from C4da neurons in larvae fed vinblastine (10 μM) or vehicle for 1 hr prior to imaging. **B-C**, Morphometric analysis reveals no significant effects of vinblastine (black bars) on total dendritic length (**A**), dendritic crossing (**B**), and axonal terminal length (**C**) compared to C4da neurons incubated with vehicle (gray bars). Bars represent average values. Error bars = SEM, n = 10 dendrites; 17–20 nerve terminals. **E**, Representative recording showing the calcium overload required to induce mPTP opening (arrow) in isolated mitochondria. **F**, Bar graph showing no significant differences in the calcium retention capacity (CRC) of isolated mitochondria treated with 10 uM vinblastine (black bar) compared to vehicle (gray bar). Bars represent average values. Error bars = SEM, n = 7 animals for each condition.

Increased pain sensitivity could result from morphological changes to the C4da neuron including alterations to the dendritic field or axonal nerve terminals [9,10]. Fig 2A shows representative fluorescent images of C4da neurons and their associated dendritic fields and axonal projections (Fig 2A, inset panels i and ii) in larvae treated with 10 uM vinblastine or vehicle for 1 hour. Quantification of neuronal morphology reveals no change in total dendritic length (Fig 2B), dendritic crossings (Fig 2C) or the axon terminal lengths (Fig 2D) consistent with vinca alkaloids having little acute effect on gross sensory neuron morphology; though, pain sensitization has already appeared by this time. These results support that the initial increase in sensitivity to pain in response to acute exposure to vinca alkaloids is largely independent of morphological changes to peripheral sensory neurons.

Previous studies have also implicated mitochondrial toxicity as the cause of CIPN [11,59,60]. To investigate the possibility that the enhanced pain sensitivity observed in response to acute exposure to vinca alkaloids is due to the direct effects of vinca alkaloids on mitochondrial health, we exposed isolated mitochondria to vinblastine and assayed calcium retention and mTPT opening, an established assay for investigating mitotoxicity [61,62]. The analyses found no difference in calcium retention or mTPT opening in mitochondria acutely incubated with vinblastine compared to controls demonstrating that vinblastine does not initially affect mitochondrial health (Fig 2E and 2F).

### Vinca alkaloids increase the excitability of C4da sensory neurons

**Fig 3:**
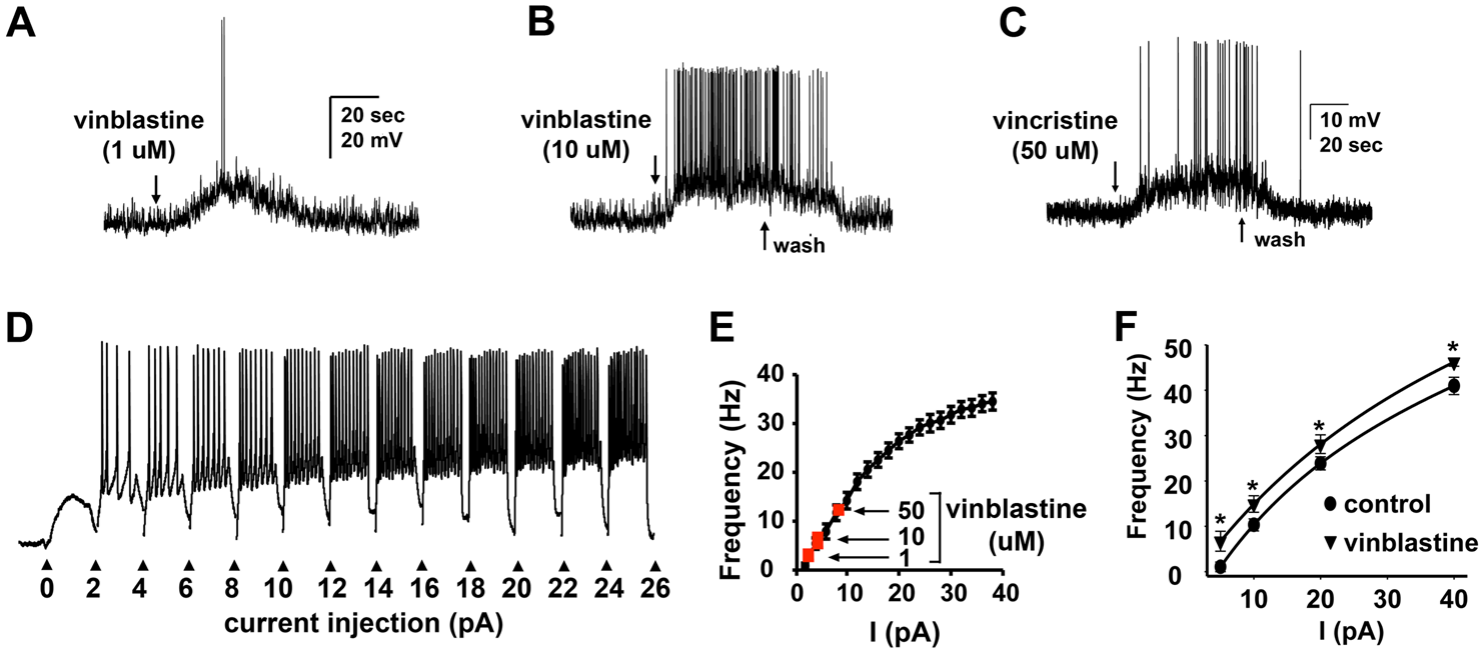
Vinca alkaloids increase the excitability of C4da sensory neurons. **A-C**, Representative traces from current-clamped C4da neurons show that exposure to vinblastine (**A, B**) or vincristine (**C**) immediately depolarizes the neuron and generates action potentials that is sensitive to the dose and presence of the drug. **D** and **E**, Current injections determine the relationship between current and firing frequency in C4da neurons (**D**). These analyses reveal that the firing frequencies resulting from vinca alkaloids (**E**) are consistent with amplitudes of the resulting current measured in voltage-clamp. Data for vinblastine (1, 10, and 50 μM) are indicated in red. Points represent average values. Error bars = SEM, n = 8–15 neurons per condition. (**F**) Graph represents the relationship between current injection and action potential frequency in the presence (arrowhead) and absence (circle) of sub-threshold concentrations of vinblastine. Each point represents the average frequency. Error bars = SEM, n = 3–5 neurons per condition. *p<0.05 determined via Student’s T-test for vinblastine vs. control for each current injection.

We next investigated the possibility that vinca alkaloids directly change the excitability of the somatosensory neurons using patch-clamp electrophysiological recordings of cultured C4da neurons challenged with vinca alkaloids [25,63,64]. We find that direct application of vinblastine and vincristine to current-clamped C4da neurons is able to rapidly drive these neurons to threshold provoking dose-dependent action potential firing (Fig 3A–3C). A frequency-current relation was generated for C4da neurons by injecting increasing amount of suprathreshold current (Fig 3D). As documented in Fig 3E, the rate at which action potentials fire in response to exposure to vinca alkaloid is consistent with the generation of an inward current evoked by vinblastine in C4da neurons (red squares) driving neuronal firing. We do not observe spontaneous action potentials in our current clamp conditions and the recordings are highly stable. We were able to observe consistent responses in current clamped C4da neurons to vinblastine concentrations as low as 10 nM. Moreover, as shown in Fig 3F, analysis of current injections in C4da neurons exposed to 1nM concentrations that fail to produce action potentials revealed a shift in the curve representing the relationship between action potential firing (frequency) versus current injections (stimuli) (Fig 3F) demonstrating that even low concentrations of vinca alkaloids that failed to elicit action potentials still had the ability to increase the excitability of C4da neurons. The increase in the hyperexcitability of C4da neurons implies an ionic mechanism for the mechanical allodynia and thermal hyperalgesia observed in larvae exposed to vinca alkaloids.

### Vinca alkaloids activate a dTrpA1-dependent depolarizing current in C4da sensory neurons

**Fig 4:**
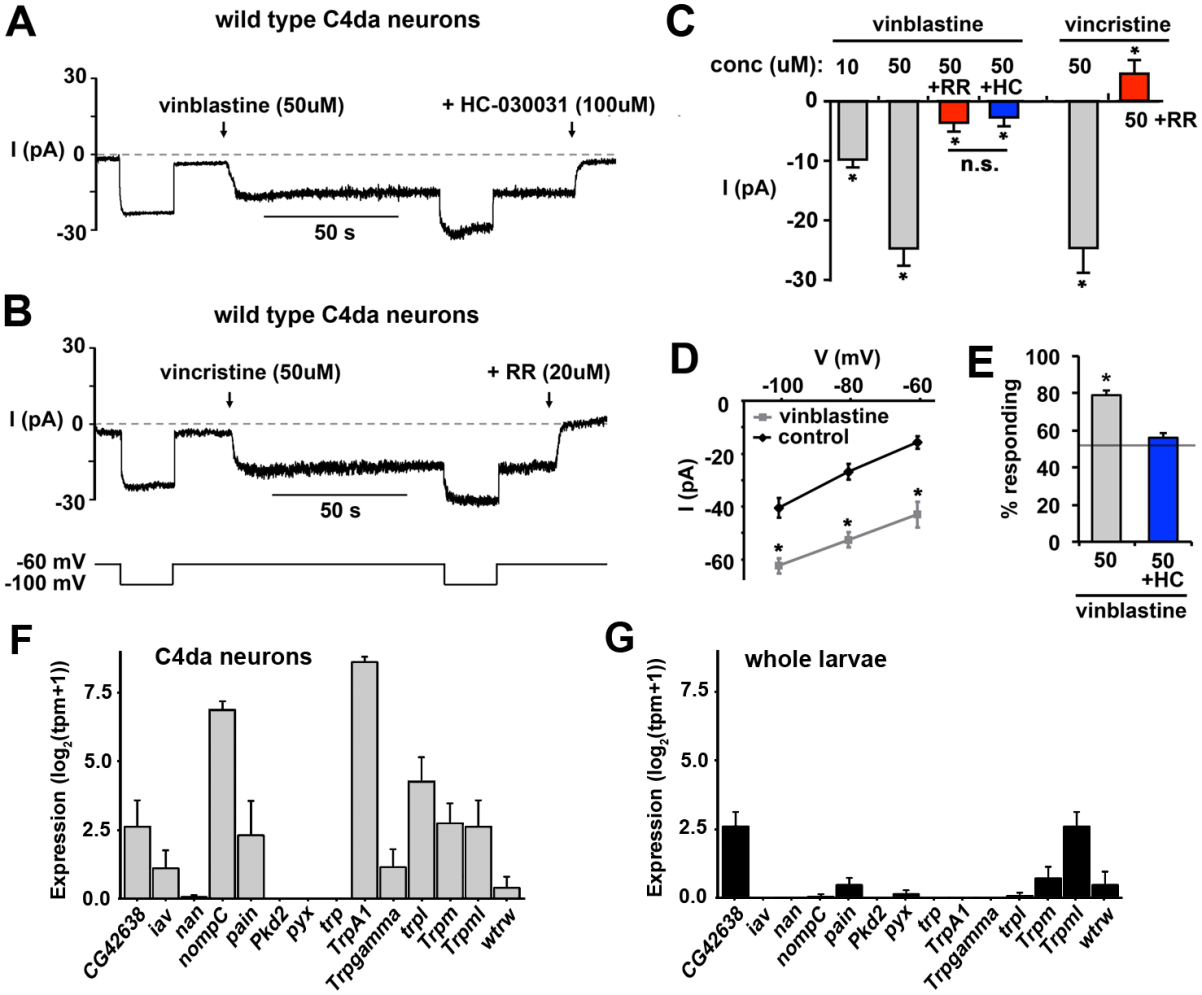
Vinca alkaloids directly excite C4da sensory neurons. **A-B**, Representative traces from voltage-clamped C4da neurons held at indicated voltages and challenged with 50 μM vinblastine (**A**) or 50 μM vincristine (**B**) reveal an immediate inward current that is sensitive to HC-030031 (**A**) and Ruthenium red (RR) (**B**). **C**, Graph represents the average values for the currents measured in voltage-clamped C4da sensory neurons resulting from the indicated treatments. * p<0.01 indicate values significantly different than all other values as determined by ANOVA. Error bars = SEM, n = 3–18 neurons per condition. **D**, Values represent the average current (I) obtained at each holding voltage (V) in C4da neurons exposed to vehicle (black line) or 50 uM vinblastine (gray line). *p<0.01 determined for each value by a Student’s T-test. Error bars = SEM, n = 3–4 neurons per condition. **E**, Graph represents the average nociceptive responses of larvae fed 50 uM Vinblastine in the absence (gray bar) or presence of 100 uM HC-030031 (blue bar). * p < 0.05 determined by Student’s T-test comparing each condition. Gray line indicates the value obtained for vehicle control larvae. Error bars = SEM, n = 160 larvae per condition. **F-G**, Graphs represent the average gene expression level of all transcripts of the indicated *Drosophila* TRP family genes in purified C4da neurons (**F**; gray bars) and in whole larvae (**G**; black bars). tpm = transcripts per million. Error bars = sem. n = 4 independent samples for each condition.

The tight spatiotemporal coupling of the effects of vinca alkaloids on excitation of C4da neurons suggested an effect of these drugs on the proteins (e.g. ion channels) that control neuronal excitability. We investigated this possible mechanism using patch-clamp electrophysiology of primary C4da neurons. We find that the direct application of vincristine (Fig 4A) and vinblastine (Fig 4B) is able to rapidly and reversibly evoke an inward current in voltage-clamped C4da neurons. This vinca-evoked current is a depolarizing Na^+^ current that is sensitive to vinca alkaloid dose (Fig 4C). This vinca-evoked current is also inhibited by both Ruthenium red (RR) (Fig 4B and 4C) and the specific TrpA1 antagonist HC-030031 (HC) supporting the role of the TrpA1 channel in the vinca response (Fig 4A–4C) [42,65]. Furthermore, analysis of the current:voltage relation in C4da neurons exposed to vinca-alkaloids is consistent with the activation of a non-voltage gated cation channel on the surface of the sensory neuron (Fig 4D). We also observe that the vinca-evoked current is very slow to adapt in the presence of drug (Fig 4A and 4B), and in all of our recordings (~5 min each) we have never observed complete desensitization of the vinca-evoked current. This could explain why we observe a persistent and robust pain sensitivity in larvae even after 24 hours of drug exposure. Together with our previous data, these results support the model that vinca alkaloids can generate a slowly-desensitizing depolarizing sodium current in the sensory neuron that is sensitive to dTrpA1 blockage. In support of a role for this current in the pain hypersensitivity observed in larvae fed vinca-alkaloids we fed larvae vinblastine that included the HC-030031 compound and found that inclusion of the HC-030031 compound blocks the resulting mechanical allodynia consistent with the vinca-evoked inward current contributing to the hypersensitivity observed in larvae fed vinca alkaloids (Fig 4E).

**Fig 5:**
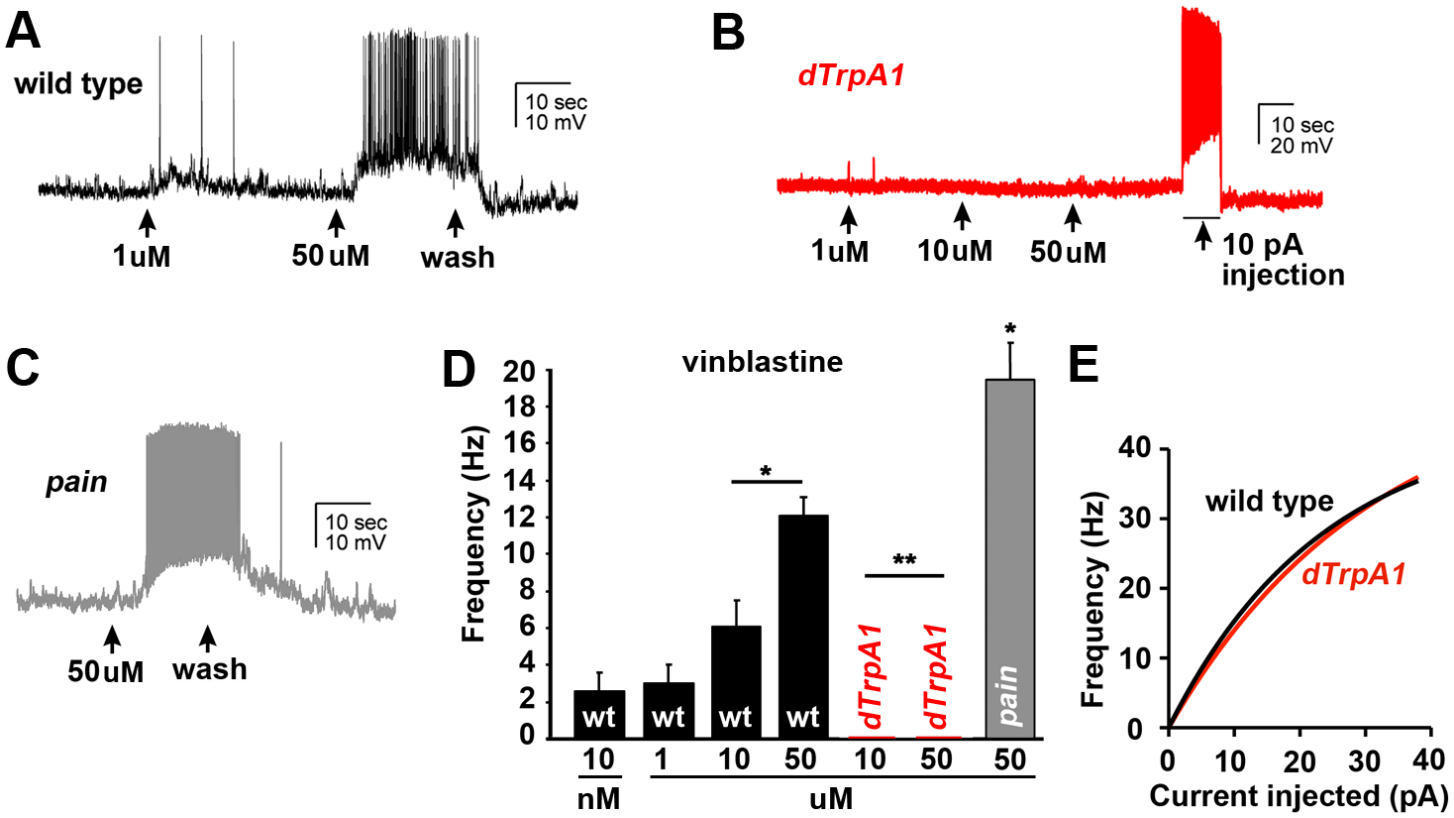
The effects of vinca alkaloids on neuron excitability are reduced in *dTrpA1* mutant C4da sensory neurons. **A-D**, Current-clamp recordings of C4da neurons reveal that the dosage effects of vinblastine on neuronal firing frequencies (**A** and **D**) are completely absent in *dTrpA1* mutant C4da neurons (**B** and **D**, red bars). In contrast, *pain* mutant C4da respond robustly to vinblastine (**C** and **D**, gray bars). **D**, Graph represents average values for action potential frequencies as a function vinblastine dose from C4da sensory neurons isolated from wild type (wt, black bars), *dTrpA1* (red bars), and *pain* (gray bars) mutants. * p<0.05, ** p<0.001 indicate values significantly different than all other values as determined by ANOVA. Error bars = SEM, n = 3–15 neurons per condition. **E**, Comparison of current injection in *dTrpA1* mutant (**B**) to wild type finds no difference in the excitability of wild type (black line) and *dTrpA1* (red line) C4da neurons.

The sensitivity of the vinca-evoked Na^+^ currents in C4da neurons to Ruthenium red and HC-030031 suggested the involvement of dTrpA1 ion channels in the effects of vinca alkaloids on the hyperexcitability of these cells [42,65]. To support the identification of dTrpA1 as the target of vinca alkaloids in C4da neurons we performed RNA-seq analysis on purified C4da neurons. Average gene expression was determined for all *Drosophila* TRP family channels and revealed that dTrpA1 is the highest expressed TRP channel in C4da neurons (Fig 4F) and shows the most enrichment within C4da neurons compared to other tissues (Fig 4G). These data are consistent with C4da neurons expressing dTrpA1 channels and support the pharmacological data suggesting that dTrpA1 is the target of vinca alkaloids during the activation of C4da neurons. To further investigate the role of dTrpA1 in the cellular response to vinca alkaloids, we investigated the effects of vinca alkaloids on C4da neuron function in *dTrpA1* mutants. In current-clamp experiments on purified C4da neurons, deletion of *dTrpA1* abolishes the concentration-dependent depolarization of C4da neuron in response to vinblastine in every neuron tested (Fig 5A, 5B and 5D). In addition to dTrpA1, a second member of the TrpA channel family, Painless (Pain), also functions in C4da neurons during nociception [21]. In contrast to *dTrpA1* mutant C4da neurons, C4da neurons isolated from *painless (pain)* mutants respond robustly to vinblastine (Fig 5C and 5D) demonstrating that the effects of vinblastine on C4da excitability are specific to dTrpA1 channels. Furthermore, analysis of firing frequency in response to current injection reveals that removal of the dTrpA1 channel from C4da sensory neurons has no effect on the excitation of these neurons. Thus, the loss of the vinca-evoked depolarization of *dTrpA1* mutant neurons is not due to a requirement of dTrpA1 channels for action potential firing in C4da sensory neurons (Fig 5B and 5E).

**Fig 6:**
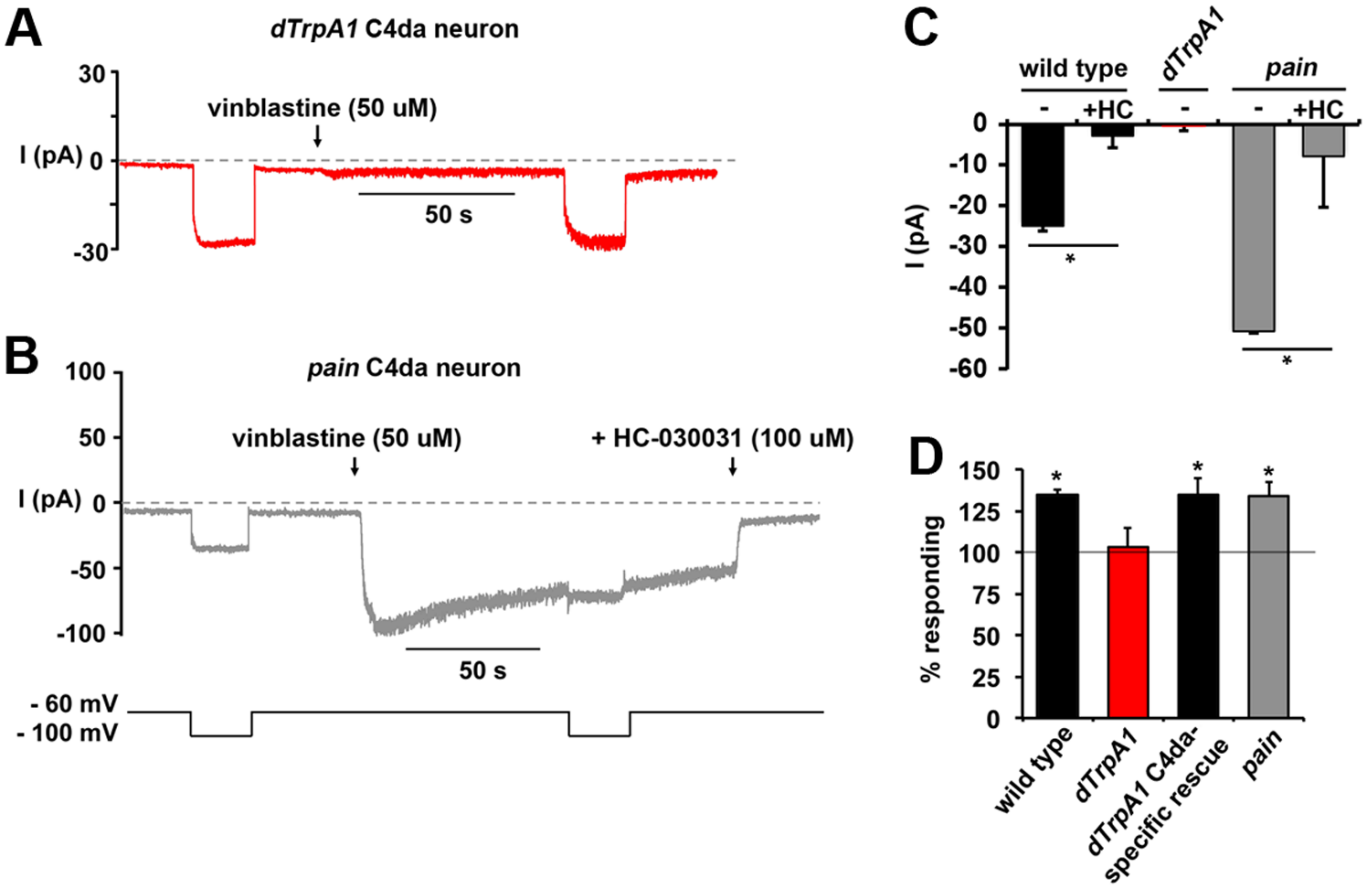
The effects of vinblastine on sensory neurons are absent in *dTrpA1* mutants. **A** and **B**, Representative traces from voltage-clamped C4da sensory neurons isolated from *dTrpA1* (**A**) or *pain* (**B**) mutants held at indicated voltages and challenged with 50 μM vinblastine. **C**, Graph represents the average values for the currents measured in voltage-clamped C4da sensory neurons from wild type (black bars; data from Fig 4C for comparison), dTrpA1 (red bar), and pain (gray) mutants incubated with 50 uM vinblastine with or without HC-030031 (+HC). * p<0.01 indicate significant difference determined by Student’s T-test. Error bars = SEM, n = 3–6 neurons per condition. **D**, Behavioral analysis of *dTrpA1* (red bars) and *pain* (gray bars) mutant larvae find that the effects of vinblastine are specifically absent in *dTrpA1* mutants and that re-expression of wild type dTrpA1 only in C4da neurons rescues this deficit in *dTrpA1* mutants. For these genetic analyses, average values were normalized to the vehicle controls assayed in parallel of the same experimental genotype. Gray line indicates vehicle (100%) values. * p < 0.05 determined by Student’s T-test of drug vs. vehicle for each genotype. Error bars = SEM, n = 160–200 larvae per condition.

To confirm the absence of the vinca-evoked inward sodium current in *dTrpA1* mutant sensory neurons, we performed voltage-clamp analysis on *dTrpA1* and *pain* mutant sensory neurons. These analyses revealed that the inward current activated by vinblastine was absent in all *dTrpA1* mutant C4da sensory neurons tested (Fig 6A and 6C). Consistent with our current-clamp analysis we observe a robust vinca-evoked inward sodium current in *pain* mutant C4da sensory neurons that were sensitive to HC-030031 (Fig 6B and 6C). Although the *pain* mutant C4da sensory neurons seem to show an enhanced desensitization immediately following application of vinblastine this vinca-evoked current in *pain* mutant C4da neurons does not completely desensitize within the time frame of our experiments, similar to what we observe for desensitization in wild type neurons. Taken together, these findings support the model that the pain hypersensitivity generated by vinca alkaloids in larvae is due to the activation of the dTrpA1 channel in C4da sensory neurons resulting in the depolarization of the neuronal membrane towards threshold, making the neuron easier to excite. In support of the notion that this electrical mechanism underlies the pain hypersensitivity observed in larvae, *dTrpA1* mutant larvae were found to lack vinblastine-sensitive mechanical allodynia (Fig 6F; red bar) compared to wild type or *pain* mutant larvae (Fig 6F; gray bar). It should be noted that we do not observe significant differences in basal mechanosensation between wild type and *dTrpA1* mutants in these experiments (data not shown). This is consistent with the previous studies indicating that the primary role of *Drosophila* dTrpA1 in C4da neurons is during thermal nociception, not mechanonociception [63,64,66]. Importantly, targeted re-expression of dTrpA1 specifically in C4da neurons rescues the vinblastine evoked mechanical allodynia in *dTrpA1* mutant larvae demonstrating that the C4da sensory neuron in the cellular loci of this molecular mechanism during pain hypersensitivity in response to vinca alkaloids (Fig 6F).

### Acute application of vinca alkaloids to sensory neurites generates thermal and mechanical hypersensitivity that is reduced in TrpA1 knock-out mice

**Fig 7:**
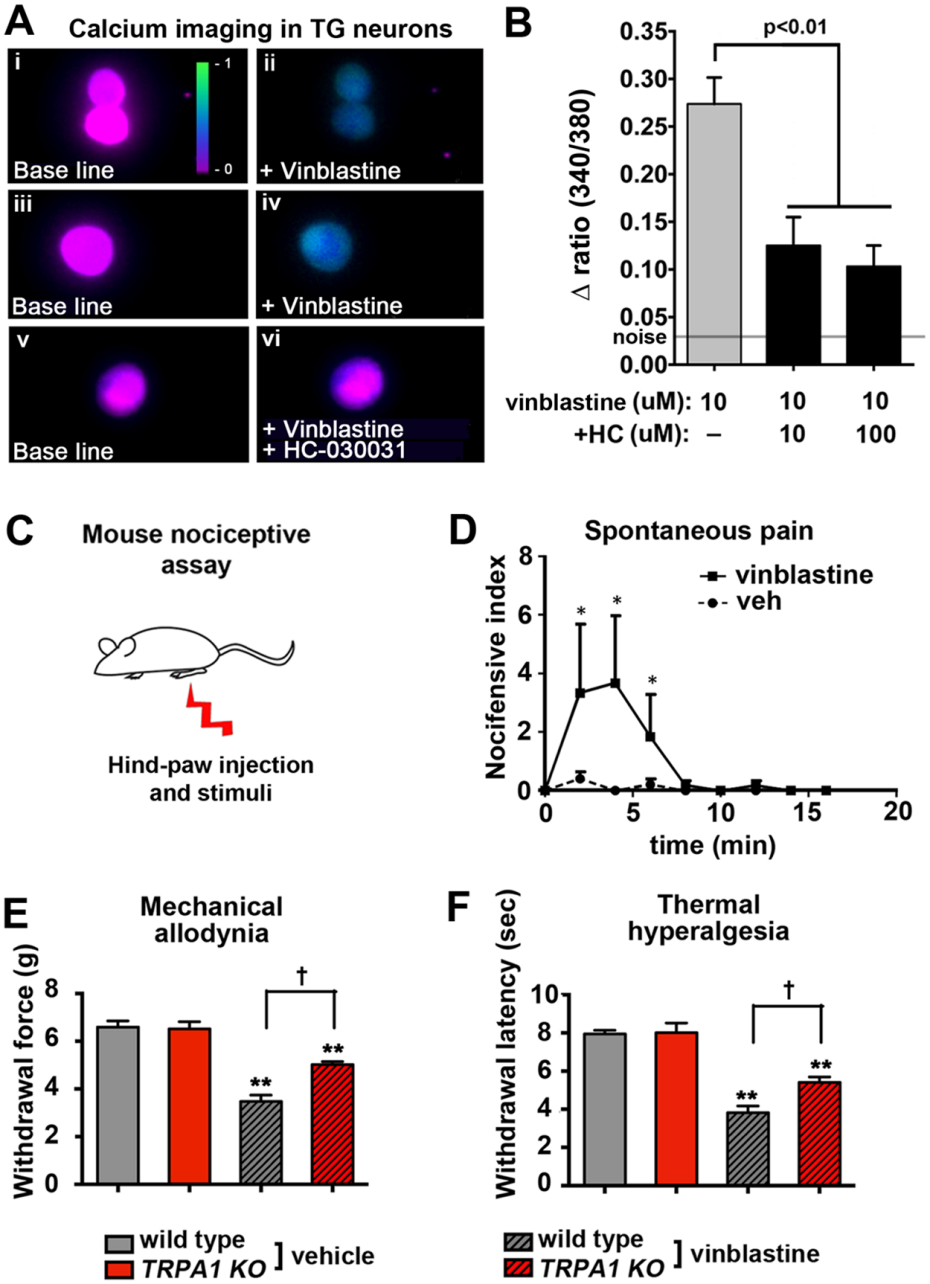
Acute exposure to vinblastine activates mammalian sensory neurons and generates pain that is reduced in TRPA1 knockout mice. **A** Representative images of Trp-V1 expressing TG neurons challenged with vinblastine (10 uM, panel ii), (100 uM, panel iv), and vinblastine + HC-030031 (10 uM; panel vi). Base line fluorescence images shown in panels i, iii, and v. **B** Graphs represent the values for the average calcium accumulation within Trp-V1 expressing TG neurons challenged with 10 uM vinblastine (gray bar) or vinblastine + HC-030031 (black bars). Error bars = SEM. n = 23–32 neurons from 4 slides per condition. P value determined using ANOVA analysis. Control noise level indicated by gray line. **C** and **D**, Single injection of vinblastine (squares) directly into the hindpaw of wild type mice (**C**) results in increased spontaneous pain compared to vehicle (circles). Values represent average duration (in seconds) in bouts of nocifensive behavior. Error bars = SEM, n = 7 animals per group. *p<0.05 determined by Student’s T-test for each point versus vehicle control. **E** and **F**, Single injection of vinblastine (striped bars) into the hindpaw results in a significant mechanical allodynia (**E**) and thermal hyperalgesia (**F**) compared to control injections (solid bars). This effect is significantly reduced in TrpA1 knock-out mice (*TRPA1 KO*, red bars). ** p<0.01 determined by Student’s T-test comparing vinblastine versus control values. † p<0.01 determined by Student’s T-test comparing wild type versus *TRPA1* mutants. Bars represent average values. Error bars = SEM, n = 7 animals per group.

*Drosophila* dTrpA1 is conserved with both mouse and human TrpA1 channels (>35% a.a. identity, >55% a.a. conserved versus mouse). Based on these similarities we predicted that mammalian sensory neurons that harbor TrpA1 channels would be activated by vinca alkaloids resulting in pain. To investigate this possibility, we first performed ratiometric calcium imaging on sensory neurons from Trigeminal ganglion (TG) where TrpA1 expressing neurons are known to also express TrpV1 [67,68]. We found that challenging of TrpV1-expressing TG neurons with 10 uM vinblastine resulted in a significant increase in intracellular calcium levels that was blocked by the inclusion of HC-030031 (Fig 7A and 7B). In these experiments, the presence of TrpV1 was verified by imaging the capsaicin response in all neurons challenged with vinblastine and only TrpV1 positive neurons were used in the generation of the average values. These calcium data are consistent with vinca alkaloids activating mammalian sensory neurons in a TrpA1-dependent fashion.

Based on our model, we predict that acute exposure of sensory neurites to vinblastine is sufficient to generate a peripheral pain phenotype via activation of TrpA1. Therefore we wanted to investigate whether the acute exposure of mammalian peripheral neurites to vinca alkaloids could also result in pain hypersensitivity in a similar TrpA1-dependent manner. To help target the distal sensory neurites that innervate the epidermis we injected the vinblastine directly into the paw of the animal (Fig 7C). We find that a single injection of vinblastine directly into the hind-paw of mice results in an immediate nocifensive response that is not observed in response to vehicle injection (Fig 7D). In wild type mice, this general nocifensive response rapidly progresses to mechanical allodynia within 15 minutes of injection (Fig 7E; gray bars) and to thermal hyperalgesia within 30 minutes of injection (Fig 7F; gray bars). Importantly, both the mechanical allodynia and thermal hyperalgesia resulting from the vinblastine injection are significantly reduced in *TRPA1* knockout mice (*TRPA1 KO*) compared to wild type mice analyzed in parallel (Fig 7B and 7C; red bars). The reduction observed in the immediate nocifensive response in TRPA1 KO mice was not significant (data not shown). These results are consistent with an evolutionarily conserved role of TrpA1 during the generation of pain hypersensitivity in response to acute vinca alkaloid exposure of sensory neurons. It should be noted that the reduction in both calcium accumulation and pain sensitivity in these mammalian experiments is only partial, suggesting the involvement of TrpA1-independent mechanisms in these responses as well.

## Discussion

In the present study, we show that the acute exposure of peripheral sensory neurons to vinca alkaloid class of anti-cancer drugs results in a robust pain syndrome that is characterized by a rapid increase in the sensitivity to both mechanical and thermal stimuli. Mutational deletion of the *TrpA1* gene significantly reduces this effect of vinca alkaloids in both mice and *Drosophila* larvae revealing an ancient and conserved requirement of TrpA1 for these acute effects of vinca alkaloids on pain hypersensitivity. Rescue studies in *Drosophila* demonstrated that the pain generated by vinca alkaloids was completely dependent upon the expression of dTrpA1 only within the C4da neuron. Patch-clamp analysis of *Drosophila* C4da sensory neurons revealed that vinca alkaloids rapidly activate a slowly-desensitizing inward depolarizing sodium current that is sensitive to the TrpA1 antagonist HC-030031 and absent in *dTrpA1* mutant C4da neurons. We also show that mammalian sensory neurons are also activated by vinca alkaloids and that activation is sensitive to HC-030031. Based on these analyses, we propose that acute exposure of nociceptors to vinca alkaloids leads to a conserved pain hypersensitivity that results from the TrpA1-dependent hyperexcitation of sensory neurons.

Our data also reveal that the effects of vinca alkaloids are not specific to modality since we observe similar effects on both thermal and mechanical nociception. It is interesting that mutations in *dTrpA1*, which have little effect on base line mechanical nociception, completely blocks the increased mechanical nociception observed in response to vinca alkaloid. One possibility is that the activation of *dTrpA1* by vinca alkaloids is simply altering the rheobase of the neuron making it more receptive to all stimuli including mechanical and thermal. In support, we observed that at low concentrations (1–10 nM), the vinca-evoked depolarizing current can reduce the threshold current of the C4da sensory neuron (i.e. rheobase) making the neurons easier to stimulate. This cellular model of pain is consistent with studies of sensory neurons in rodents where pain-causing doses of vinca-alkaloids increased spontaneous firing of sensory neurons [6]. It was also shown that an unspecified sub-set of peripheral sensory nociceptors within the rat dorsal root ganglion (DRG) become more excitable after 7–10 days of exposure to paclitaxel, although a role for TrpA1 was not investigated [7]. Taken together, these data support the model that activation of TrpA1 in peripheral nociceptors is an important early event during the onset of CIPN in response to vinca alkaloids.

Supporting an early role for TrpA1 activation during the onset of sensory symptoms during CIPN, a single administration of members the platinum and proteasome inhibitor classes of anti-cancer drugs into mice results in mechanical and cold allodynia within 12 hours of administration that is reduced in *TRPA1* mutants or by blocking studies with the HC-030031 TrpA1 antagonist [42,43]. A similar requirement for TrpA1 function during the hyperalgesia resulting from paclitaxel and cyclophosphamide were also demonstrated using the HC-030031 antagonist [41,44]. Importantly, patients treated with oxaplatinum drugs report a robust cold allodynia in the legs and throat and other symptoms arising during drug infusion or immediately thereafter [69–71]. These studies (and ours) provide evidence that TrpA1 channels in sensory neurons are important targets of a broad range of anti-cancer drugs and that activation of these channels represents and early event during the pathogenesis of sensory symptoms associated with CIPN.

It should be noted that in the mouse, inhibition of TrpA1 genetically or pharmacologically only partially reduced the effects of vinblastine on nociception. This suggests that other sensory transduction mechanisms also contribute to the pain generated by vinblastine in mammals. Rodent studies support that some anti-cancer drugs are able to generate pain syndromes via the actions of other TRP family members including TRPV1 and TRPV4 [8,72,73]. These data highlight that the pain resulting from anti-cancer drugs is complex and likely due to the actions of multiple Trp family channels, including TrpA1.

### Activation of TrpA1 by vinca alkaloids

Previous studies have shown that TrpA1 channels can be activated via indirect mechanisms resulting from the change in inflammation, oxidative stress, and mitochondrial toxicity associated with chronic exposure to anti-cancer drugs [8,11,41,42,74,75]. These cellular pathologies then drive changes in TrpA1 channel function or channel expression resulting in increased pain sensitivity [7,42] [76]. Although our data does not demonstrate direct activation of TrpA1, we believe that the timescale of activation (within seconds) observed in C4da neurons exposed to vinca alkaloids is not consistent with indirect mechanisms such as changes in gene expression, altered mitochondrial function, or alteration of the channel composition. In support, we don’t see an acute effect of vinca alkaloids on mitochondrial calcium handling. This time scale would accommodate coupled signaling systems, such as PLC signaling, that are known to regulate Trp channel activity [28]. Importantly, our model also does not rule out an important contribution of these pathological mechanisms to CIPN. In fact, we would expect an increase in oxidative stress and mitochondrial dysfunction as a result of chronic hyperexcitation of the sensory neuron due to TrpA1 activation.

The direct activation of TrpA1 channels could occur via binding of anti-cancer drugs to previously identified TrpA1 protein domains. For example, TrpA1 channels contain highly conserved cysteines that mediate the activation of TrpA1 via the direct binding of toxic electrophiles to this domain [30,33]. Although recently it was shown that citronella, which also activates a slowly desensitizing TrpA1-dependent depolarizing current in gustatory neurons does not require these cysteines for activation [77]. Another possibility is that anti-cancer drugs activate TrpA1 via the conserved ankyrin repeat domain (ARD), which consists of a large number of tandem ankyrin repeats located in the N-terminus of all TrpA1 channels and required for normal TrpA1 function including responses to noxious chemicals [78] [31,40,79]. Although a role for the ARs in mechanosensation by TrpA1 channels has yet to be established, recent data from another *Drosophila* TrpA channel NompC demonstrate that the ARs located in the N terminus of NompC are necessary and sufficient for mechanical gating of the channel [80]. In larvae, NompC has been shown to function in C3da neurons during gentle touch and locomotion [81,82]. Importantly, Zhang and colleagues demonstrated that these ARs link NompC to the microtubule cytoskeleton and that an intact microtubule cytoskeleton is required for mechanical gating of NompC [80]. This mechanism is reminiscent of the microtubule-dependent gating of TrpV1 in osmosensory neurons during cell shrinkage [83]. Thus it is also possible that the activation of TrpA1 by vinca alkaloids is the result of a direct microtubule-based gating mechanism due to the effects of vinca alkaloids on the microtubule cytoskeleton.

### Relevance to CIPN

Is it possible that this cellular mechanism could contribute to the effects of vinca alkaloids on human chemotherapy patients? In terms of pain, humans with a gain-of-function mutation in the *TRPA1* gene develop Familial Episodic Pain Syndrome (FEPS), a pain syndrome associated with enhanced sensitivity to noxious mechanical and chemical stimuli [84]. Analysis of this mutation using HEK239 cells reveals a large increase in the inward current observed in response to channel activation compared to wild type channels demonstrating that TrpA1 channel activation in sensory neurons can contribute to pain in humans [84]. Interestingly, this mutation also causes a change in the current:voltage relationship of the transgenic TrpA1 channel that is very similar to what we observe to the vinca-evoked current in C4da neurons supporting the feasibility of our TrpA1-dependent mechanism in humans. Finally, TrpA1 channels are known to function in chemical nociception across species by activating sensory neurons exposed to noxious chemicals providing an evolutionary argument for the conservation of this mechanism [27,28,85]. Taken together, these studies support that enhanced sensory neuron excitability via TrpA1 activation represents a candidate mechanism underlying the pathogenesis of pain hypersensitivity in response to potentially a broad number of anti-cancer drugs.

An important consideration is the effective concentrations of vinca alkaloids surrounding the sensory neurites. Although it is difficult to know for certain what the concentrations of vinca alkaloids are near the sensory neurites during chemotherapy, concentrations of 0.8 ng/ml (~ 0.9 nM) have been measured in cerebrospinal fluid 24 hours following treatment in some patients [86]. Vinca concentrations in plasma are usually much higher than the CSF and can spike to as high as 100 ng/ml (108 nM) within minutes following a single low dose of vincristine (2 mg/m^2^) [87]. We find that concentrations to 10 nM effectively elicit neuronal firing within seconds of exposure in our cultures and even the lower concentrations of vinblastine (to 1 nM) that fail to elicit action potentials are nonetheless sufficient to lower the rheobase of the exposed neuron resulting in a neuron that is easier to excite. Thus, it is possible that even low concentrations of vinca alkaloids can activate this sensory mechanism in patients during chemotherapy treatments. Our analyses in mice and flies demonstrate that the effects of vinca alkaloids on sensory neuron function and pain hypersensitivity are conserved evolutionarily across diverse phyla, revealing an ancient and conserved role for the TrpA1 channel in the cellular response to anti-cancer drugs.
